# Supplementary material for: Comparative Genomic Analysis Discloses Differential Distribution of Antibiotic Resistance Determinants between Worldwide Strains of the Emergent ST213 Genotype of Salmonella Typhimurium
Source: Antibiotics (Basel). 2022 Jul 9;11(7):925. doi: 10.3390/antibiotics11070925 (PMC9312005; doi:10.3390/antibiotics11070925)
Supplement: Supplementary file 1 [file antibiotics-11-00925-s001.zip › antibiotics-1801853-supplementary.pdf]

Supplementary Table S1. Data of the analyzed genomes in the present work.

No.	Sample	Country	Year	Source[1]	Accession No./Assembly Barcode
1	AUS01H_2017	Australia	2017	Human	SRR14143603
2	AUS02H_2018	Australia	2018	Human	SRR14145155
3	AUS03H_2006	Australia	2006	Human	SRR5090724
4	AUS04_2021	Australia	2021	ND/Others	SAL_PC2750AA_AS
5	BEL01H_2020	Belgium	2020	Human	ERR4775375
6	CAN01H_2009	Canada	2009	Human	SRR12712089
7	CAN02H_2018	Canada	2018	Human	SRR13426370
8	CAN03H_2011	Canada	2011	Human	SRR13605059
9	CAN04H_2008	Canada	2008	Human	SRR13769784
10	CAN05H_2017	Canada	2017	Human	SRR13827060
11	CAN06H_2017	Canada	2017	Human	SRR13849853
12	CAN07H_2017	Canada	2017	Human	SRR13952785
13	CAN08H_2017	Canada	2017	Human	SRR13952800
14	CAN09H_2018	Canada	2018	Human	SRR14102894
15	CAN10H_2018	Canada	2018	Human	SRR14102906
16	CAN11H_2018	Canada	2018	Human	SRR14767203
17	CAN19H_2016	Canada	2016	Human	SRR15242716
18	CAN12H_2017	Canada	2017	Human	SRR15242717
19	CAN13H_2015	Canada	2015	Human	SRR15242758
20	CAN14H_2016	Canada	2016	Human	SRR15242930
21	CAN15H_2014	Canada	2014	Human	SRR15242943
22	CAN16H_2014	Canada	2014	Human	SRR15242946
23	CAN17H_2008	Canada	2008	Human	SAL_KC5843AA_AS
24	CAN18H_2010	Canada	2010	Human	SAL_KC5864AA_AS
25	CAN21H_2018	Canada	2018	Human	SAL_OC2346AA_AS
26	CAN20H_2017	Canada	2017	Human	SAL_RC8451AA_AS
27	DEN01H_2002	Denmark	2002	Human	ERR2719681
28	DEN02H_2011	Denmark	2011	Human	ERR2729121
29	DEN04H_2017	Denmark	2017	Human	ERR2849935
30	DEN03H_2009	Denmark	2009	Human	ERR2729149
31	DEN05H_2009	Denmark	2009	Human	ERR3060821
32	DEN06H_2008	Denmark	2008	Human	ERR3901222
33	FRA01H_2019	France	2019	Human	SAL_MC3552AA_AS
34	FRA02H_2019	France	2019	Human	SAL_MC3509AA_AS
35	IND01F_2011	India	2011	Food	SRR1107438
36	IND02F_2010	India	2010	Food	SRR1300736
37	IND03F_2015	India	2015	Food	SRR2029592
38	IRE01H_2007	Ireland	2007	Human	ERR2693630
39	IRE02H_2016	Ireland	2016	Human	SAL_OA1032AA_AS
40	MEX26H_2003	Mexico	2003	Human	ERR2693961
41	MEX38H_2004	Mexico	2004	Human	ERR2719629

42	MEX40H_2003	Mexico	2003	Human	ERR2719652
43	MEX01B_2004	Mexico	2004	Bovine	ERR2719833
44	MEX56R_2019	Mexico	2019	Water/River	SRR11016193
45	MEX57R_2019	Mexico	2019	Water/River	SRR11028646
46	MEX58R_2019	Mexico	2019	Water/River	SRR11028649
47	MEX05D_2019	Mexico	2019	Water/Dam	SRR11029506
48	MEX06D_2019	Mexico	2019	Water/Dam	SRR11029518
49	MEX07D_2019	Mexico	2019	Water/Dam	SRR11029520
50	MEX59R_2019	Mexico	2019	Water/River	SRR11029522
51	MEX08D_2019	Mexico	2019	Water/Dam	SRR11029523
52	MEX60R_2019	Mexico	2019	Water/River	SRR11029526
53	MEX09D_2019	Mexico	2019	Water/Dam	SRR11029528
54	MEX10D_2019	Mexico	2019	Water/Dam	SRR11029530
55	MEX11F_2005	Mexico	2005	Food	SRR6010020
56	MEX12F_2004	Mexico	2004	Food	SRR9017255
57	MEX53_2004	Mexico	2004	ND/Others	SRR9099710
58	MEX13F_2004	Mexico	2004	Food	SRR9102687
59	MEX54_2004	Mexico	2004	ND/Others	SRR9102691
60	MEX55_2004	Mexico	2004	ND/Others	SRR9102693
61	MEX24H_2003	Mexico	2003	Human	ERR2693572
62	MEX25H_2005	Mexico	2005	Human	ERR2693575
63	MEX27H_2005	Mexico	2005	Human	ERR2693974
64	MEX61S_2003	Mexico	2003	Swine	ERR2693980
65	MEX28H_2003	Mexico	2003	Human	ERR2693981
66	MEX29H_2003	Mexico	2003	Human	ERR2710803
67	MEX30H_2004	Mexico	2004	Human	ERR2710805
68	MEX31H_2002	Mexico	2002	Human	ERR2710819
69	MEX32H_2003	Mexico	2003	Human	ERR2710835
70	MEX33H_2004	Mexico	2004	Human	ERR2710854
71	MEX34H_2003	Mexico	2003	Human	ERR2710856
72	MEX35H_2003	Mexico	2003	Human	ERR2710865
73	MEX36H_2003	Mexico	2003	Human	ERR2710869
74	MEX37H_2004	Mexico	2004	Human	ERR2710884
75	MEX39H_2003	Mexico	2003	Human	ERR2719641
76	MEX41H_2004	Mexico	2004	Human	ERR2719677
77	MEX42H_2004	Mexico	2004	Human	ERR3482042
78	MEX62S_2003	Mexico	2003	Swine	ERR3482056
79	MEX43H_2002	Mexico	2002	Human	ERR3482104
80	MEX44H_2007	Mexico	2007	Human	ERR3901480
81	MEX45H_2005	Mexico	2005	Human	ERR3901484
82	MEX46H_2004	Mexico	2004	Human	ERR3901491
83	MEX63S_2004	Mexico	2004	Swine	ERR3901497
84	MEX47H_2005	Mexico	2005	Human	ERR3901500
85	MEX64S_2003	Mexico	2003	Swine	ERR3901506

86	MEX04C_2003	Mexico	2003	Chicken	ERR4399209
87	MEX48H_2003	Mexico	2003	Human	ERR4400263
88	MEX49H_2004	Mexico	2004	Human	ERR4400784
89	MEX66B_2004	Mexico	2004	Bovine	SAL_UA1479AA_AS
90	MEX67H_2002	Mexico	2002	Human	SAL_UA1639AA_AS
91	MEX65B_2003	Mexico	2003	Bovine	SAL_UA1633AA_AS
92	MEX50H_2005	Mexico	2005	Human	GCF_001006525
93	MEX51H_2011	Mexico	2011	Human	GCF_001293505
94	MEX02B_2004	Mexico	2004	Bovine	GCF_009497835
95	MEX03B_2004	Mexico	2004	Bovine	GCF_009497855
96	MEX52H_2007	Mexico	2007	Human	GCF_011304815
97	NET01H_2009	Netherlands	2009	Human	ERR4400176
98	POR01F_1965	Portugal	1965	Food	SAL_IA6848AA_AS
99	POR02F_1967	Portugal	1967	Food	SAL_IA6884AA_AS
100	THA02H_2003	Thailand	2003	Human	SRR1106424
101	THA03H_2002	Thailand	2002	Human	SRR1840679
102	THA01C_2019	Thailand	2019	Chicken	SRR14011968
103	UK02H_2004	United Kingdom	2004	Human	ERR2719837
104	UK16H_2012	United Kingdom	2012	Human	SRR1645887
105	UK17H_2014	United Kingdom	2014	Human	SRR1957801
106	UK18H_2014	United Kingdom	2014	Human	SRR1957825
107	UK20H_2014	United Kingdom	2014	Human	SRR1965680
108	UK21_2014	United Kingdom	2014	ND/Others	SRR1967397
109	UK22H_2014	United Kingdom	2014	Human	SRR1967622
110	UK23H_2014	United Kingdom	2014	Human	SRR1968141
111	UK25H_2015	United Kingdom	2015	Human	SRR1969768
112	UK27_2015	United Kingdom	2015	ND/Others	SRR3286840
113	UK28H_2015	United Kingdom	2015	Human	SRR3322115
114	UK29H_2017	United Kingdom	2017	Human	SRR5584700
115	UK30H_2015	United Kingdom	2015	Human	SRR6062349
116	UK31H_2015	United Kingdom	2015	Human	SRR6074595
117	UK32H_2015	United Kingdom	2015	Human	SRR6111080
118	UK33H_2018	United Kingdom	2018	Human	SRR7090670
119	UK34H_2016	United Kingdom	2016	Human	SRR7159857
120	UK35H_2018	United Kingdom	2018	Human	SRR7215972
121	UK36H_2016	United Kingdom	2016	Human	SRR7286838
122	UK38H_2018	United Kingdom	2018	Human	SRR7402355
123	UK39H_2017	United Kingdom	2017	Human	SRR7408361
124	UK40H_2017	United Kingdom	2017	Human	SRR7444204
125	UK41H_2018	United Kingdom	2018	Human	SRR7451170
126	UK42H_2017	United Kingdom	2017	Human	SRR7516730
127	UK43H_2018	United Kingdom	2018	Human	SRR7828239
128	UK44H_2018	United Kingdom	2018	Human	SRR7828437
129	UK45H_2018	United Kingdom	2018	Human	SRR7850686

130	UK46H_2018	United Kingdom	2018	Human	SRR7867043
131	UK47H_2018	United Kingdom	2018	Human	SRR7879573
132	UK48H_2018	United Kingdom	2018	Human	SRR7910405
133	UK49H_2018	United Kingdom	2018	Human	SRR7962214
134	UK50H_2017	United Kingdom	2017	Human	SRR8548892
135	UK51H_2017	United Kingdom	2017	Human	SRR8553861
136	UK52F_2016	United Kingdom	2016	Food	SRR8658156
137	UK53H_2019	United Kingdom	2019	Human	SRR9054604
138	UK54H_2019	United Kingdom	2019	Human	SRR9261071
139	UK55H_2019	United Kingdom	2019	Human	SRR9719498
140	UK04H_2000	United Kingdom	2000	Human	ERR2693688
141	UK01H_2001	United Kingdom	2001	Human	ERR2693713
142	UK03H_2003	United Kingdom	2003	Human	ERR4399105
143	UK05H_2019	United Kingdom	2019	Human	SRR10519589
144	UK06H_2019	United Kingdom	2019	Human	SRR10519615
145	UK07H_2019	United Kingdom	2019	Human	SRR10561173
146	UK08H_2019	United Kingdom	2019	Human	SRR10600365
147	UK09H_2019	United Kingdom	2019	Human	SRR10810736
148	UK10H_2020	United Kingdom	2020	Human	SRR10900167
149	UK11H_2020	United Kingdom	2020	Human	SRR11041608
150	UK12H_2021	United Kingdom	2021	Human	SRR13485880
151	UK13_2018	United Kingdom	2018	ND/Others	SRR15366175
152	UK14H_2016	United Kingdom	2016	Human	SRR15370189
153	UK15H_2021	United Kingdom	2021	Human	SRR16134724
154	UK19H_2014	United Kingdom	2014	Human	SRR1958128
155	UK24H_2014	United Kingdom	2014	Human	SRR1968540
156	UK26H_2014	United Kingdom	2014	Human	SRR1970253
157	UK37H_2016	United Kingdom	2016	Human	SRR7359015
158	UK64Wa_2009	United Kingdom	2009	Wild Animal	SRR17893191
159	UK63H_2019	United Kingdom	2019	Human	SAL_SB9289AA_AS
160	UK60H_2016	United Kingdom	2016	Human	SAL_AB5684AA_AS
161	UK61H_2017	United Kingdom	2017	Human	SAL_BB4395AA_AS
162	UK62H_2017	United Kingdom	2017	Human	SAL_JB1106AA_AS
163	UK56F_2016	United Kingdom	2016	Food	SAL_CB6634AA_AS
164	UK57_2018	United Kingdom	2018	ND/Others	SAL_ZA4977AA_AS
165	UK58_2018	United Kingdom	2018	ND/Others	SAL_BB3548AA_AS
166	UK59F_2016	United Kingdom	2016	Food	SAL_CB6630AA_AS
167	US01S_1999	United States	1999	Swine	SRR10063260
168	US05S_2006	United States	2006	Swine	SRR1212306
169	US06S_2003	United States	2003	Swine	SRR1272873
170	US07B_2020	United States	2020	Bovine	SRR12899007
171	US08S_2000	United States	2000	Swine	SRR13024097
172	US09_2020	United States	2020	ND/Others	SRR13221214
173	US13H_2007	United States	2007	Human	SRR1840738

174	US14S_2006	United States	2006	Swine	SRR2532791
175	US15S_2015	United States	2015	Swine	SRR2648752
176	US16H_2012	United States	2012	Human	SRR3097408
177	US17H_2011	United States	2011	Human	SRR3097427
178	US18H_2010	United States	2010	Human	SRR3097562
179	US19H_2011	United States	2011	Human	SRR3097569
180	US20H_2015	United States	2015	Human	SRR3232066
181	US21H_2015	United States	2015	Human	SRR3232068
182	US22H_2015	United States	2015	Human	SRR3233166
183	US23H_2014	United States	2014	Human	SRR4420901
184	US24H_2016	United States	2016	Human	SRR5081545
185	US25H_2017	United States	2017	Human	SRR5336307
186	US26H_2016	United States	2016	Human	SRR5361040
187	US27H_2017	United States	2017	Human	SRR5418841
188	US28H_2012	United States	2012	Human	SRR5573114
189	US29S_2017	United States	2017	Swine	SRR5576032
190	US30H_2017	United States	2017	Human	SRR5605850
191	US31H_2017	United States	2017	Human	SRR5628564
192	US32H_2017	United States	2017	Human	SRR5876908
193	US34H_2017	United States	2017	Human	SRR6334793
194	US35H_2014	United States	2014	Human	SRR6491048
195	US36H_2014	United States	2014	Human	SRR6685032
196	US37H_2015	United States	2015	Human	SRR6685073
197	US38H_2017	United States	2017	Human	SRR6750589
198	US39H_2018	United States	2018	Human	SRR6879627
199	US40_2018	United States	2018	Human	SRR6885440
200	US41P_1957	United States	1957	Poultry	SRR7084742
201	US42H_2012	United States	2012	Human	SRR8435362
202	US43H_2011	United States	2011	Human	SRR8435708
203	US44H_2010	United States	2010	Human	SRR8435716
204	US45H_2010	United States	2010	Human	SRR8435722
205	US46H_2010	United States	2010	Human	SRR8597284
206	US47H_2017	United States	2017	Human	SRR8832720
207	US48S_2006	United States	2006	Swine	SRR949413
208	US02S_1999	United States	1999	Swine	SRR10063300
209	US03H_2007	United States	2007	Human	SRR1133231
210	US04S_2002	United States	2002	Swine	SRR11457455
211	US10_2021	United States	2021	ND/Others	SRR15257871
212	US11S_2003	United States	2003	Swine	SRR1539626
213	US12B_2021	United States	2021	Bovine	SRR16976151
214	US33C_1988	United States	1988	Canine	SRR6311308
215	US49B_2019	United States	2019	Bovine	SAL_BC0558AA_AS
216	US51S_2002	United States	2002	Swine	SAL_EC9748AA_AS
217	US52S_2002	United States	2002	Swine	SAL_EC9744AA_AS

218	US53S_2002	United States	2002	Swine	SAL_FC0153AA_AS
219	US54S_2003	United States	2003	Swine	SAL_FC0279AA_AS
220	US50B_2020	United States	2020	Bovine	SAL_KC7851AA_AS

Note: [1] ND, non-determined

Supplementary Table S2. Data of the ST213 genotype draft genomes obtained from Enterobase[1].

No.	Sample	Accession No. /Assembly Barcode	Genome Length[2]	No. Contigs	N50	Largest Contig	AvgContigLen[3]
1	AUS04_2021	SAL_PC2750AA_AS	4841509	187	59979	244544	25890
2	CAN17H_2008	SAL_KC5843AA_AS	5009731	64	334376	881602	78277
3	CAN18H_2010	SAL_KC5864AA_AS	4963062	61	376801	802111	81361
4	CAN21H_2018	SAL_OC2346AA_AS	4887527	80	276895	684657	61094
5	CAN20H_2017	SAL_RC8451AA_AS	4967271	72	394003	881829	68989
6	FRA01H_2019	SAL_MC3552AA_AS	4920430	69	177888	570640	71310
7	FRA02H_2019	SAL_MC3509AA_AS	4902545	138	66077	313196	35525
8	IRE02H_2016	SAL_OA1032AA_AS	4827944	67	223598	698801	72058
9	MEX66B_2004	SAL_UA1479AA_AS	5002191	53	301495	698272	94380
10	MEX67H_2002	SAL_UA1639AA_AS	5083011	65	394092	881604	78200
11	MEX65B_2003	SAL_UA1633AA_AS	5109605	63	393714	881420	81104
12	MEX50H_2005	GCF_001006525	5190370	8	4886081	4886081	648796
13	MEX51H_2011	GCF_001293505	5088186	4	4809574	4809574	1272046
14	MEX02B_2004	GCF_009497835	5096576	4	4837346	4837346	1274144
15	MEX03B_2004	GCF_009497855	5060451	6	4764173	4764173	843408
16	MEX52H_2007	GCF_011304815	5113293	5	4863742	4863742	1022658
17	POR01F_1965	SAL_IA6848AA_AS	4880496	57	376801	683163	85622
18	POR02F_1967	SAL_IA6884AA_AS	4842310	73	195060	498144	66333
19	UK63H_2019	SAL_SB9289AA_AS	4825282	64	198811	635641	75395
20	UK60H_2016	SAL_AB5684AA_AS	4872229	149	107756	324799	32699
21	UK61H_2017	SAL_BB4395AA_AS	4762122	144	108315	327435	33070
22	UK62H_2017	SAL_JB1106AA_AS	4779598	107	87158	392483	44669
23	UK56F_2016	SAL_CB6634AA_AS	5064442	92	225728	451048	55048
24	UK57_2018	SAL_ZA4977AA_AS	4935995	72	223707	699340	68555
25	UK58_2018	SAL_BB3548AA_AS	4876068	68	351396	746587	71706
26	UK59F_2016	SAL_CB6630AA_AS	4867593	74	246550	630437	65778
27	US49B_2019	SAL_BC0558AA_AS	4891355	113	91629	380793	43286
28	US51S_2002	SAL_EC9748AA_AS	4926650	102	282557	843495	48300
29	US52S_2002	SAL_EC9744AA_AS	4935341	154	162197	357102	32047
30	US53S_2002	SAL_FC0153AA_AS	4896274	95	260444	424211	51539
31	US54S_2003	SAL_FC0279AA_AS	4796988	68	376786	843658	70543
32	US50B_2020	SAL_KC7851AA_AS	4775207	63	376487	777577	75796

Notes: [1] Most of the genomes were obtained with Ilumina platform, except GCF_001006525, with PacBio; 454; Ilumina GAIIx; GCF_001293505, with PacBio RS; GCF_009497835, with PacBio RSII; GCF_009497855 and GCF_01130481, with Oxford Nanopore MiniION. [2]Obtained by TORMES; [3]AvgContigLen = Average Contig Length.

Supplementary Table S3. Data of the ST213 genotype draft genomes assembled in this work.

No.	Sample	Accession No.	Genome Length	No. Contigs	N50	Largest Contig	AvgContigLen[1]
1	AUS01H_2017	SRR14143603	5005729	103	713682	224020	48599
2	AUS02H_2018	SRR14145155	4783219	66	506183	195937	72473
3	AUS03H_2006	SRR5090724	4868786	145	376140	117021	33577
4	BEL01H_2020	ERR4775375	4914470	45	287411	699084	109210
5	CAN01H_2009	SRR12712089	4959284	125	417030	118966	39674
6	CAN02H_2018	SRR13426370	4888923	107	611176	223641	45690
7	CAN03H_2011	SRR13605059	4939010	91	444440	179928	54274
8	CAN04H_2008	SRR13769784	5139077	163	431220	100074	31528
9	CAN05H_2017	SRR13827060	4737356	86	464039	190984	55085
10	CAN06H_2017	SRR13849853	4744496	104	376801	190984	45620
11	CAN07H_2017	SRR13952785	4870261	94	405182	258927	51811
12	CAN08H_2017	SRR13952800	4741452	100	579139	282799	47414
13	CAN09H_2018	SRR14102894	4889645	94	690747	282799	52017
14	CAN10H_2018	SRR14102906	4977996	94	690745	376801	52957
15	CAN11H_2018	SRR14767203	4986889	87	881786	376801	57320
16	CAN12H_2017	SRR15242717	5044018	88	880527	282999	57318
17	CAN13H_2015	SRR15242758	4925887	71	880465	301268	69378
18	CAN14H_2016	SRR15242930	4888133	79	881419	393714	61875
19	CAN15H_2014	SRR15242943	5090233	79	881601	377931	64433
20	CAN16H_2014	SRR15242946	4937121	79	881417	393714	62495
21	CAN19H_2016	SRR15242716	4926622	232	192201	56233	21235
22	DEN01H_2002	ERR2719681	4984481	182	750054	255860	27387
23	DEN02H_2011	ERR2729121	4857281	162	520999	213830	29983
24	DEN03H_2009	ERR2729149	5148477	53	223258	690860	97141
25	DEN04H_2017	ERR2849935	4871803	116	506473	164553	41998
26	DEN05H_2009	ERR3060821	5144940	51	274130	809358	100881
27	DEN06H_2008	ERR3901222	5009163	49	243737	717899	102227
28	IND01F_2011	SRR1107438	4899621	114	699340	255941	42979
29	IND02F_2010	SRR1300736	4844434	97	699340	198572	49942
30	IND03F_2015	SRR2029592	4882663	189	699340	213930	25834
31	IRE01H_2007	ERR2693630	4821095	34	323073	549620	141796
32	MEX01B_2004	ERR2719833	5073584	213	628512	282373	23819
33	MEX04C_2003	ERR4399209	5047407	67	301232	628447	75334
34	MEX05D_2019	SRR11029506	4847992	83	819000	246039	58409
35	MEX06D_2019	SRR11029518	4851815	83	628510	218638	58455
36	MEX07D_2019	SRR11029520	4849629	82	818999	282777	59141
37	MEX08D_2019	SRR11029523	4851671	82	628678	223363	59166
38	MEX09D_2019	SRR11029528	4852917	88	880793	223363	55146
39	MEX10D_2019	SRR11029530	4851087	75	804935	223363	64681
40	MEX11F_2005	SRR6010020	4984709	97	690745	297919	51388
41	MEX12F_2004	SRR9017255	5149322	156	698621	183055	33008
42	MEX13F_2004	SRR9102687	5077540	123	684646	196723	41280

43	MEX24H_2003	ERR2693572	5090293	57	246638	498032	89303
44	MEX25H_2005	ERR2693575	5053940	81	238479	498021	62394
45	MEX26H_2003	ERR2693961	5226363	172	862980	376701	30385
46	MEX27H_2005	ERR2693974	5184205	63	301234	578153	82288
47	MEX28H_2003	ERR2693981	5055607	51	340697	818845	99129
48	MEX29H_2003	ERR2710803	5103298	53	376541	818964	96288
49	MEX30H_2004	ERR2710805	5011626	45	335267	818986	111369
50	MEX31H_2002	ERR2710819	5042977	58	282644	880998	86947
51	MEX32H_2003	ERR2710835	5214099	59	335267	595479	88374
52	MEX33H_2004	ERR2710854	5062610	57	282961	628497	88817
53	MEX34H_2003	ERR2710856	5121709	53	376541	819028	96636
54	MEX35H_2003	ERR2710865	5060644	53	376541	880997	95483
55	MEX36H_2003	ERR2710869	5042111	70	137483	366728	72030
56	MEX37H_2004	ERR2710884	4890507	41	228871	577382	119280
57	MEX38H_2004	ERR2719629	5096019	236	819186	240337	21593
58	MEX39H_2003	ERR2719641	5178225	59	246700	595279	87766
59	MEX40H_2003	ERR2719652	5115286	230	819354	283231	22240
60	MEX41H_2004	ERR2719677	5024458	64	201110	796774	78507
61	MEX42H_2004	ERR3482042	5031586	42	276732	819018	119799
62	MEX43H_2002	ERR3482104	5033740	68	196368	628357	74025
63	MEX44H_2007	ERR3901480	5033592	42	282876	819396	119847
64	MEX45H_2005	ERR3901484	5036881	50	276486	866946	100737
65	MEX46H_2004	ERR3901491	5036640	54	265342	819203	93271
66	MEX47H_2005	ERR3901500	5124857	65	223076	577790	78843
67	MEX48H_2003	ERR4400263	5060979	60	246672	690117	84349
68	MEX49H_2004	ERR4400784	5090047	60	280191	628306	84834
69	MEX53_2004	SRR9099710	4994658	98	881418	298344	50965
70	MEX54_2004	SRR9102691	5020626	150	684612	279416	33470
71	MEX55_2004	SRR9102693	5224092	121	881602	377276	43174
72	MEX56R_2019	SRR11016193	4996578	78	733742	301490	64058
73	MEX57R_2019	SRR11028646	4862823	81	628512	196238	60034
74	MEX58R_2019	SRR11028649	4861711	77	716177	201315	63139
75	MEX59R_2019	SRR11029522	4852841	77	628510	223363	63023
76	MEX60R_2019	SRR11029526	4853195	79	690305	223363	61432
77	MEX61S_2003	ERR2693980	5166477	46	301234	595396	112314
78	MEX62S_2003	ERR3482056	5001881	55	301234	696959	90943
79	MEX63S_2004	ERR3901497	5012726	44	301227	819125	113925
80	MEX64S_2003	ERR3901506	5079411	50	276578	690121	101588
81	NET01H_2009	ERR4400176	4961200	41	273107	745306	121004
82	THA01C_2019	SRR14011968	4981734	37	333625	745908	134641
83	THA02H_2003	SRR1106424	4875802	108	545399	141530	45146
84	THA03H_2002	SRR1840679	4863904	113	361630	175502	43043
85	UK01H_2001	ERR2693713	4816370	45	213554	698735	107030
86	UK02H_2004	ERR2719837	5136441	234	740641	282373	21950

87	UK03H_2003	ERR4399105	4993894	43	301104	610177	116137
88	UK04H_2000	ERR2693688	4860468	34	377353	681673	142954
89	UK05H_2019	SRR10519589	4809723	55	178073	506316	87449
90	UK06H_2019	SRR10519615	4808838	57	152759	506467	84365
91	UK07H_2019	SRR10561173	4868036	54	201920	627869	90148
92	UK08H_2019	SRR10600365	4848636	58	145075	377070	83597
93	UK09H_2019	SRR10810736	4810321	56	164066	506081	85898
94	UK10H_2020	SRR10900167	4812824	56	178078	377063	85943
95	UK11H_2020	SRR11041608	4807025	66	145071	330486	72833
96	UK12H_2021	SRR13485880	4709598	46	222922	547828	102382
97	UK13_2018	SRR15366175	4846205	61	178073	376487	79445
98	UK14H_2016	SRR15370189	4919495	54	213661	480882	91101
99	UK15H_2021	SRR16134724	4867726	60	168525	524400	81128
100	UK16H_2012	SRR1645887	5008330	123	536760	188144	40718
101	UK17H_2014	SRR1957801	4915187	222	367624	111333	22140
102	UK18H_2014	SRR1957825	4727624	138	495228	120281	34258
103	UK19H_2014	SRR1958128	4769042	148	51118	195382	32223
104	UK20H_2014	SRR1965680	4775349	87	520939	149344	54889
105	UK21_2014	SRR1967397	4769372	181	221189	58289	26350
106	UK22H_2014	SRR1967622	4972525	91	438474	171329	54643
107	UK23H_2014	SRR1968141	4716217	100	247110	130554	47162
108	UK24H_2014	SRR1968540	4914777	93	94543	194596	52847
109	UK25H_2015	SRR1969768	4765403	99	438583	213775	48135
110	UK26H_2014	SRR1970253	4911862	113	104190	267122	43467
111	UK27_2015	SRR3286840	4775822	72	506599	192094	66330
112	UK28H_2015	SRR3322115	4860815	98	506045	134356	49600
113	UK29H_2017	SRR5584700	4774223	113	255530	91445	42249
114	UK30H_2015	SRR6062349	4817828	87	506704	187070	55377
115	UK31H_2015	SRR6074595	4823317	88	506430	178183	54810
116	UK32H_2015	SRR6111080	4819431	87	506337	170134	55395
117	UK33H_2018	SRR7090670	4852645	78	398413	195744	62213
118	UK34H_2016	SRR7159857	4931502	91	442640	278051	54192
119	UK35H_2018	SRR7215972	4826826	93	506048	145156	51901
120	UK36H_2016	SRR7286838	4921493	81	627762	196194	60759
121	UK37H_2016	SRR7359015	4913045	119	84743	231872	41286
122	UK38H_2018	SRR7402355	4717468	58	818704	171817	81335
123	UK39H_2017	SRR7408361	4820570	86	320870	186932	56053
124	UK40H_2017	SRR7444204	4781053	64	506241	178182	74703
125	UK41H_2018	SRR7451170	4864252	95	506058	182615	51202
126	UK42H_2017	SRR7516730	4785033	84	506520	187009	56964
127	UK43H_2018	SRR7828239	4865649	63	404856	195736	77232
128	UK44H_2018	SRR7828437	4857716	109	320863	110301	44566
129	UK45H_2018	SRR7850686	4820031	85	506337	187070	56706
130	UK46H_2018	SRR7867043	4857683	86	470066	149343	56484

131	UK47H_2018	SRR7879573	4856791	82	324616	178183	59229
132	UK48H_2018	SRR7910405	4861250	78	506337	178183	62323
133	UK49H_2018	SRR7962214	4778772	69	320863	185520	69257
134	UK50H_2017	SRR8548892	4717030	57	497880	222576	82754
135	UK51H_2017	SRR8553861	4818260	94	320863	143281	51258
136	UK52F_2016	SRR8658156	4862720	84	628038	196210	57889
137	UK53H_2019	SRR9054604	4970061	79	506338	149529	62912
138	UK54H_2019	SRR9261071	4910924	78	324477	186932	62960
139	UK55H_2019	SRR9719498	4982417	78	628037	190277	63877
140	UK64Wa_2009	SRR17893191	4894539	60	178070	506499	81575
141	US01S_1999	SRR10063260	5024874	134	777921	184452	37499
142	US02S_1999	SRR10063300	4860499	191	42375	186524	25447
143	US03H_2007	SRR1133231	5397470	93	149553	428033	58037
144	US04S_2002	SRR11457455	4890263	78	158492	414669	62695
145	US05S_2006	SRR1212306	4837178	83	843679	377608	58279
146	US06S_2003	SRR1272873	4973359	120	539654	224390	41444
147	US07B_2020	SRR12899007	4954094	131	267822	126841	37817
148	US08S_2000	SRR13024097	4995418	85	683993	393714	58769
149	US09_2020	SRR13221214	5013520	69	690746	376800	72659
150	US10_2021	SRR15257871	4998408	55	222653	880812	90880
151	US11S_2003	SRR1539626	5012142	51	257511	683073	98277
152	US12B_2021	SRR16976151	4912376	150	62781	212495	32749
153	US13H_2007	SRR1840738	5116122	117	867275	238695	43727
154	US14S_2006	SRR2532791	4905444	85	843495	376786	57711
155	US15S_2015	SRR2648752	5035448	139	628524	196325	36226
156	US16H_2012	SRR3097408	4883365	189	320444	130556	25837
157	US17H_2011	SRR3097427	4941374	156	189650	79226	31675
158	US18H_2010	SRR3097562	5079692	118	604635	171818	43048
159	US19H_2011	SRR3097569	5019108	124	442288	195736	40476
160	US20H_2015	SRR3232066	5001087	109	684716	224308	45881
161	US21H_2015	SRR3232068	5001291	110	512661	224904	45466
162	US22H_2015	SRR3233166	5004137	117	587415	301893	42770
163	US23H_2014	SRR4420901	4895682	140	881603	398300	34969
164	US24H_2016	SRR5081545	4953506	111	690746	394010	44626
165	US25H_2017	SRR5336307	4939187	93	827916	384085	53109
166	US26H_2016	SRR5361040	5040741	82	628500	282141	61472
167	US27H_2017	SRR5418841	5320793	136	1321794	653515	39123
168	US28H_2012	SRR5573114	5029191	86	881418	394010	58478
169	US29S_2017	SRR5576032	5095906	89	982743	223102	57257
170	US30H_2017	SRR5605850	5095277	165	513631	255131	30880
171	US31H_2017	SRR5628564	5073878	100	687841	344084	50738
172	US32H_2017	SRR5876908	4895999	115	690745	321169	42573
173	US33C_1988	SRR6311308	4899797	72	139294	363992	68052
174	US34H_2017	SRR6334793	4961916	89	690745	276882	55751

175	US35H_2014	SRR6491048	4983159	188	268654	69487	26506
176	US36H_2014	SRR6685032	4840297	76	699340	224196	63688
177	US37H_2015	SRR6685073	5155149	137	881602	282799	37628
178	US38H_2017	SRR6750589	4967988	91	587538	301490	54593
179	US39H_2018	SRR6879627	4881798	178	639984	91060	27425
180	US40_2018	SRR6885440	5018417	78	714139	256424	64338
181	US41P_1957	SRR7084742	4800131	87	735022	376801	55173
182	US42H_2012	SRR8435362	4876948	73	690746	282799	66807
183	US43H_2011	SRR8435708	5028636	136	454104	109679	36975
184	US44H_2010	SRR8435716	5104904	126	515104	282800	40515
185	US45H_2010	SRR8435722	5098439	98	603403	253501	52024
186	US46H_2010	SRR8597284	4849920	80	690747	301486	60624
187	US47H_2017	SRR8832720	5108363	134	583607	192331	38122
188	US48S_2006	SRR949413	4908411	92	690758	376786	53352

Note: [1] AvgContigLen = Average Contig Length
